# Association between sugar-sweetened beverage consumption frequency and muscle strength: results from a sample of Chinese adolescents

**DOI:** 10.1186/s12889-023-15987-z

**Published:** 2023-05-30

**Authors:** Yunjie Zhang, Pan Xu, Yongjing Song, Nan Ma, Jinkui Lu

**Affiliations:** 1 College of Education and Sports Sciences, Yangtze University, Hubei Jingzhou, 434020 P. R. China; 2 School of Preschool Education, Shangrao Preschool Education College, Jiangxi Shangrao, 334000 P. R. China; 3 College of physical education and health, Shanghai Lixin University of Accounting and Finance, 201209 Shanghai, P. R. China; 4 School of Physical Education, Shangrao Normal University, Jiangxi Shangrao, 334000 P. R. China

**Keywords:** Adolescents, Sugar-sweetened beverage consumption, Muscle strength, Cross-sectional survey

## Abstract

**Background:**

Although sugar-sweetened beverage consumption has become an important and widespread concern, there are few studies on the association between sugar-sweetened beverage consumption frequency and muscle strength in Chinese adolescents. The objective of this study was to analyze the association between sugar-sweetened beverage consumption frequency and muscle strength in Chinese adolescents.

**Methods:**

A stratified whole-group sampling method was used to survey 25,893 adolescents aged 13–15 years old in China for sugar-sweetened beverage consumption frequency and muscle strength for grip strength and standing long jump. The subjects’ basic information, body mass index (BMI), and covariates were investigated. The association between sugar-sweetened beverage consumption frequency and muscle strength was analyzed by multivariate logisitc regression analysis.

**Results:**

The proportions of Chinese adolescents who consumed sugar-sweetened beverage ≥ 3 times/week, 1–2 times/week, and < 1 time/week were 12.23%, 52.79%, and 34.98%, respectively. The differences in sugar-sweetened beverage consumption frequency were statistically significant when compared across gender, parental education, duration of physical activity, snacks, and mode of commuting to school (*χ*^2^ values = 228.570, 51.422, 275.552, 3165.656, 10.988, *P* < 0.01). Logistic regression analysis showed that overall Chinese adolescents with sugary drinks 1–2 times/week (*OR* = 1.207, 95% *CI*:1.132–1.287) and sugary drinks ≥ 3 times/week (*OR* = 1.771, 95% *CI*:1.611–1.947) were associated with lower muscle strength compared to sugary drinks < 1 time/week showed a positive association (*P* < 0.01). The same trend was found for boys and girls.

**Conclusion:**

Chinese adolescents’ sugar-sweetened beverage consumption is common, and high-frequency sugar-sweetened beverage consumption is associated with lower muscle strength. In the future, we should control the use of sugar-sweetened beverages and increase muscular strength training in Chinese adolescents to promote healthy growth.

## Introduction

Research has found that muscle strength is an important determinant of physical health [1]. Low muscle mass and low muscle strength are important contributors to adverse health problems such as falls and disability, and are also important contributing factors to metabolic dysfunction in adolescents, affecting cognitive development, bone growth and overall health during adolescence [2]. Inadequate muscle strength can also have an impact on gross and fine motor performance in adolescents and even negatively affect the future development of osteoporosis and sarcopenia [3, 4]. A study on adolescent grip strength showed that from 1967 to 2017, adolescent grip strength improved in overall trend, with countries such as the United States and Italy showing an upward trend, while some countries such as China and Belgium showed a downward or stable trend [5]. In addition, an analysis of one-handed static tension reports from Chinese and US populations also showed that US subjects exhibited greater muscle strength than Chinese subjects [6]. It has also been confirmed that the COVID-19 pandemic also had a negative impact on muscle strength status in adolescents, especially in boys much more than in girls [7]. A survey showed that Chinese adolescents had a 23.89% decrease in pull-up performance compared to pre-pandemic COVID-19 [8]. It is evident that muscle strength is important for healthy adolescent development and is influenced by various factors [9]. The literature shows that genetics [10], age [11], chronic diseases [12], lifestyle [13], daily diet [14], and physical activity [15] are all important factors that affect adolescent muscle strength. In addition, many epidemiological surveys also show that with the continuous improvement of lifestyle, the amount of sugar-sweetened beverage consumption among adolescents continues to increase and becomes an important factor affecting physical and mental health.

In terms of sugar-sweetened beverage consumption, middle-income countries are significantly higher than those at higher income levels [16]. United States in recent years sugar-sweetened beverage consumption has declined, but consumption levels are still higher than the United States Dietary Guidelines and WHO recommendations [17]. 54% of adolescents in middle- and low-income countries consume sugary drinks at least once a day [18]. The majority (87.6%) of adolescents in China consume sugary drinks, with a consumption level of 205.4 ml/day per person [19]. There are also surveys showing that China produced more than 180 million tons of various sugary drinks in 2017, 440 times more than in 1992 [20]. This shows that the consumption of sugary drinks is at a high level. A growing number of studies have shown that the consumption of sugary beverages poses a significant health risk and has been shown to be positively associated with circulatory and digestive diseases and all-cause mortality [21]. In addition, the continued increase in sugar-sweetened beverage consumption can lead to an increase in the prevalence of obesity, diabetes, osteoporosis, cardiometabolic diseases, and rectal adenomas in adolescents [22–26]. Studies have also confirmed that excessive consumption of sugary drinks can lead to changes in skeletal muscle mitochondria, which can have a negative impact on muscle strength [27]. It is evident that sugary drinks are an important factor influencing muscle strength in adolescents and a significant causal factor in the persistent decline of muscle strength in adolescents. However, the findings were not consistent with regard to the relationship between sugary drinks and muscle strength. It was found that no significant association was found between sugary drinks and muscle function [28].

The comprehensive literature shows that studies on the relationship between sugar-sweetened beverages and muscle strength in adolescents in developed countries are increasing year by year, and there are inconsistent findings. While sugar-sweetened beverage consumption continues to increase in developing countries, especially in China, it is unclear whether the increase in sugar-sweetened beverage consumption affects adolescents’ muscle strength. Given the importance of muscle strength for healthy adolescent development and the continued increase in adolescent sugar-sweetened beverage consumption, it is necessary to investigate and study the frequency of sugar-sweetened beverage consumption and muscle strength in Chinese adolescents. To this end, we tested the association between sugar-sweetened beverage consumption frequency and muscle strength in 25,893 adolescents aged 13–15 years in China and analyzed the association between the two. We aimed to provide reference and help for healthy diet and muscle strength promotion and intervention in adolescents.

## Materials and methods

### Participants and procedures

**Fig. 1:**
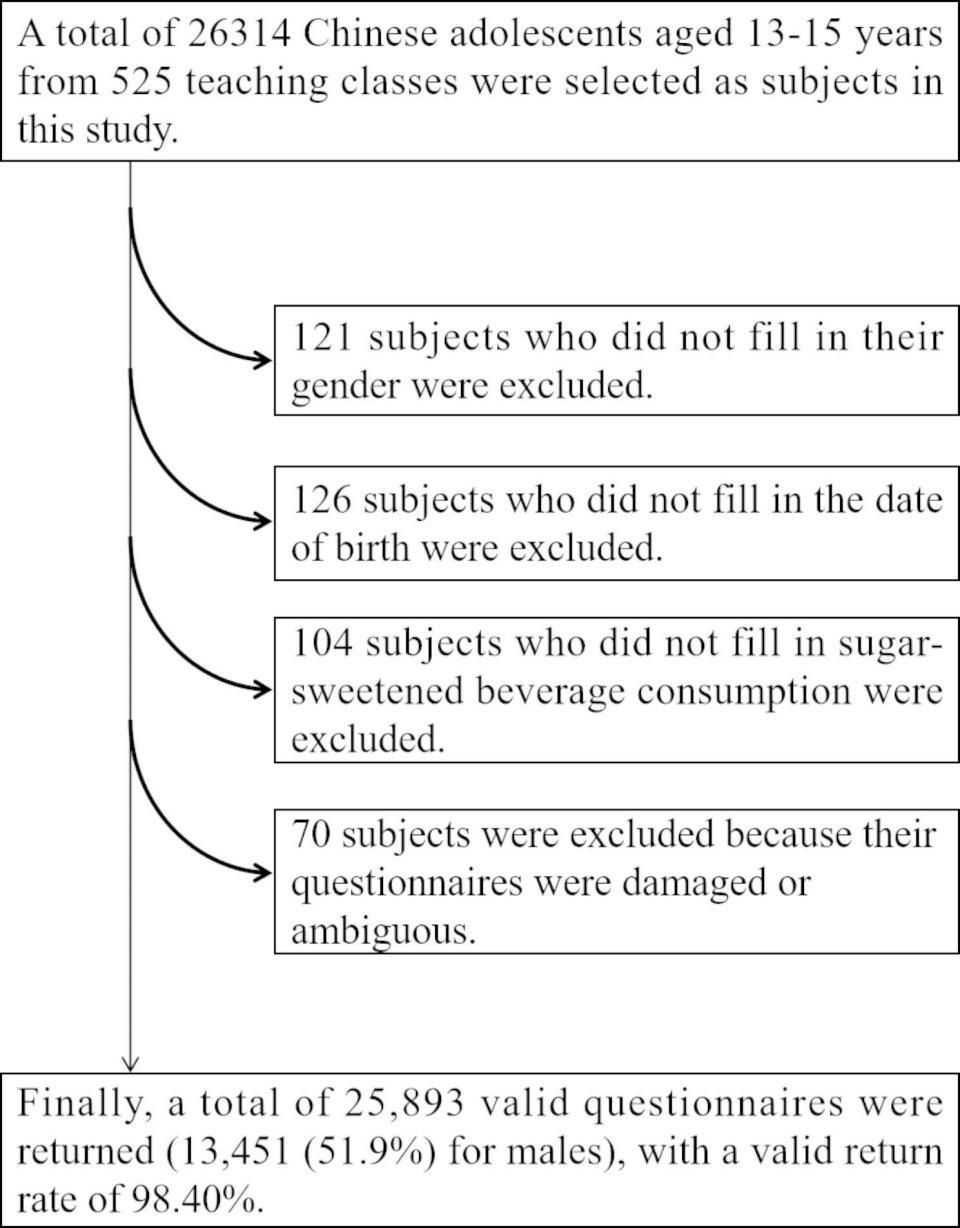
Sampling process of Chinese adolescent subjects

A stratified whole-group sampling method was used to draw the subjects. The specific steps were as follows: First, according to the geographical distribution of each province, Liaoning, Henan, Fujian, Hainan, Sichuan, Jiangxi, and Xinjiang in China were used as the survey areas. Second, each region selected a total of five secondary schools in the east-west, north-south, and central directions according to the geographical distribution of secondary schools as the survey schools. Third, each school was sampled in classes, and five teaching classes were randomly selected in whole groups for each grade level. A total of 26,314 adolescents in 525 teaching classes were selected for this study. After excluding 421 adolescents who were missing due to demographically important information after the survey, a total of 25,893 valid questionnaires were returned (13,451 boys, 51.9%), with a valid return rate of 98.40%. The specific subject extraction process is shown in Fig. 1.

This study investigation followed the ethical principles of the Declaration of Helsinki of the World Medical Association. Written informed consent was signed by the student’s parents and myself, respectively, prior to the start of this study investigation. This study was approved by the Human Ethics Committee of Shangrao Normal University (2018R-0219).

### Survey of basic demographic information

The survey of basic demographic information included subjects’ age, gender, school, class, and parents’ education, etc. Subjects were asked to conduct the questionnaire according to their actual situation. Parental education was filled in according to the highest education of one of the fathers or mothers, and was divided into three levels: elementary school and below, junior high school or high school, and college and above. In order to protect students’ personal privacy, the survey was filled in the form of coding.

### Sugar-sweetened beverage consumption frequency

The frequency of sugar-sweetened beverage consumption in the past week was investigated on the basis of relevant data and was classified as ≥ 3 times/week, 1–2 times/week, and < 1 time/week [29]. Each time, the calculation is done with a daily bottle of about 300 ml. The types of sugary drinks include sports drinks, juice drinks, coffee drinks, carbonated drinks and other aspects.

### Muscle strength test

The muscle strength was evaluated by grip strength, which reflects the muscle strength of upper limbs, and standing long jump performance, which reflects the muscle strength of lower limbs. In order to comprehensively evaluate the muscle strength level of the subjects, the grip strength and standing long jump scores were standardized according to age and gender, and the muscle strength index was obtained by summing the z-scores of both. Muscle strength index = Z_grip strength_+Z_standing long jump_. The muscle strength index was divided into percentiles according to age and gender, and those below the P25th percentile were defined as those with low muscle strength, while those above the P25th percentile were defined as those with normal muscle strength [30]. Muscle strength was tested according to the testing methods and instruments required by the China National Student Physical Health Survey [31].

### Investigation of covariates

The oblique variables in this study included indicators such as mode of transportation to and from school, frequency of snack intake, duration of physical activity, and BMI, in addition to basic demographic information. The calculation of BMI was performed based on height and weight. Height and weight were tested according to the testing instruments and methods required by the student physical fitness standards, and both height and weight were accurate to one decimal point [32]. Transportation to and from school was divided into positive and negative modes of transportation, with positive modes of transportation including walking or bicycling and negative modes including taking the subway, bus, private car transportation, and electric car. The frequency of snack intake was divided into three categories: <1 time/week, 1–2 times/week, and ≥ 3 times/week. The duration of physical exercise was mainly calculated as the time of medium-intensity physical activity in the past 7 days, and the average daily time of medium-intensity physical activity was calculated and divided into three categories: <30 min/day, 30–60 min/day, and > 60 min/day.

### Quality control

The survey of this study was conducted by trained graduate students, and the purpose and requirements of the survey were explained to the students before the survey, and the survey was conducted after receiving written informed consent from the students themselves. When the questionnaire was returned, the subjects were asked to fill in the missing and wrong entries in time to ensure the validity of the survey. The survey process was conducted under the guidance of the survey staff, and questions were answered promptly. The questionnaires were distributed on the spot and collected on the spot.

### Statistical analysis

Continuous variables were expressed as mean and standard deviation (M ± SD). The representation of count variables was expressed as a percentage.

The comparison of each variable among Chinese adolescents with different sugary drinks was performed by one-way ANOVA or chi-square test. A chi-square test was used to compare the detection rates of different frequencies of sugar-sweetened beverage consumption on muscle strength index levels in Chinese adolescents. The association between sugar-sweetened beverages and muscle strength was performed by multiple logistic regression analysis. Whether Chinese adolescents had a low muscle strength index was used as the dependent variable, and the frequency of sugar-sweetened beverage consumption was used as the independent variable. Crude models were not adjusted for relevant confounding variables, and only the association between sugar-sweetened beverage consumption and muscle strength was analyzed. Model 1 adjusted for age and parental education. Model 2 adjusted for school commuting pattern, snack intake, physical activity duration, and BMI based on model 1.

The analysis of this study was conducted using SPSS25.0(SPSS Inc., Chicago, IL, USA) software with *α* = 0.05 as a two-sided test level.

## Results

Table 1 shows that 25,893 Chinese adolescents aged 13–15 years were surveyed and tested for sugary drink consumption and muscle strength in this study. The mean age of the subjects was (14.06 ± 0.82) years. The proportions of Chinese adolescents with sugar-sweetened beverage consumption ≥ 3 times/week, 1–2 times/week, and < 1 time/week were 12.23% (3166/25,893), 52.79% (13,670/25,893), and 34.98% (9057/25,893), respectively.

**Table 1:** Comparison of different types of sugar-sweetened beverage consumption among Chinese adolescents (% or M ± SD)

Category	Sugar-sweetened beverage consumption			Total	*χ*^2^/*F*-value	*p* -value
	≥ 3 times/week	1 ~ 2 times/week	<1 times/week			
Number of people	3166	13,670	9057	25,893		
Age	14.14 ± 0.81	14.05 ± 0.82	14.05 ± 0.82	14.06 ± 0.82	17.714	<0.001
**Sex**						
Male	1944(14.45)	7289(54.19)	4218(31.36)	13,451	228.570	<0.001
Female	1222(9.82)	6381(51.29)	4839(38.89)	12,442		
**Parental Education**						
elementary school and below	200(12.19)	868(52.89)	573(34.92)	1641	51.422	<0.001
junior high or high school	2141(11.73)	9870(54.08)	6241(34.19)	18,252		
college and above	825(13.75)	2932(48.87)	2243(37.38)	6000		
**Duration of physical exercise**						
< 30 min/day	1669(14.71)	5658(49.86)	4020(35.43)	11,347	275.552	<0.001
30–60 min/day	1219(11.46)	6014(56.55)	3401(31.98)	10,634		
> 60 min/day	278(7.11)	1998(51.07)	1636(41.82)	3912		
**Snacks**						
< 1 time/week	233(5.21)	1530(34.19)	2712(60.60)	4475	3165.656	<0.001
1–2 times/week	1141(7.73)	8925(60.45)	4699(31.83)	14,765		
≥ 3 times/week	1792(26.94)	3215(48.32)	1646(24.74)	6653		
**Mode of transportation to and from school**						
Positive Approach	1404(12.11)	6009(51.83)	4181(36.06)	11,594	10.988	0.004
Negative way	1762(12.32)	7661(53.58)	4876(34.1)	14,299		
Height	166.39 ± 8.79	164.95 ± 8.6	164.48 ± 8.52	164.96 ± 8.61	58.043	<0.001
Weight	56.58 ± 11.81	54.06 ± 10.98	53.35 ± 10.61	54.12 ± 11.00	102.333	<0.001
Body mass index	20.37 ± 3.66	19.77 ± 3.21	19.63 ± 3.14	19.80 ± 3.25	61.702	<0.001

The results of this study showed that the differences in sugar-sweetened beverage consumption among Chinese adolescents were statistically significant when compared across gender, parental education, duration of physical activity, snacking, and mode of commuting to school (*χ*^2^ values = 228.570, 51.422, 275.552, 3165.656, and 10.988, *p* < 0.01). Significant differences were also found when comparing Chinese adolescents with different sugary drinks in terms of height, weight, and body mass index (*F*-value = 58.043, 102.333, 61.702, *p* < 0.001).

Table 2 shows that the differences in the detection rates of different muscle strength indices among Chinese adolescents with different sugar-sweetened beverage consumption frequency were statistically significant when compared in boys, girls, and overall (χ^2^ values = 65.455, 60.175, 123.518, *P* < 0.001). Overall, the percentage of Chinese adolescents with higher frequency of sugar-sweetened beverage consumption with a muscle index ≥ P25 was lower, at 11.1%.

**Table 2:** Comparison of different sugar-sweetened beverage consumption Chinese adolescents’ muscle strength index levels (%)

Sex	Sugar-sweetened beverage	Number of people	Muscle strength index		Total	*χ*^2^-value	*p* -value
			≥P25	<P25			
**Male**	≥ 3times/week	1944	1333(13.2)	611(18.2)	1944(14.5)	65.455	<0.001
	1-2times/week	7289	5458(54.1)	1831(54.4)	7289(54.2)		
	<1times/week	4218	3297(32.7)	921(27.4)	4218(31.4)		
**Female**	≥ 3times/week	1222	815(8.7)	407(13.1)	1222(9.8)	60.175	<0.001
	1-2times/week	6381	4771(51.1)	1610(51.8)	6381(51.3)		
	<1times/week	4839	3746(40.1)	1093(35.1)	4839(38.9)		
**Total**	≥ 3times/week	3166	2148(11.1)	1018(15.7)	3166(12.2)	123.518	<0.001
	1-2times/week	13,670	10,229(52.7)	3441(53.2)	13,670(52.8)		
	<1times/week	9057	7043(36.3)	2014(31.1)	9057(35.0)		

**Fig. 2:**
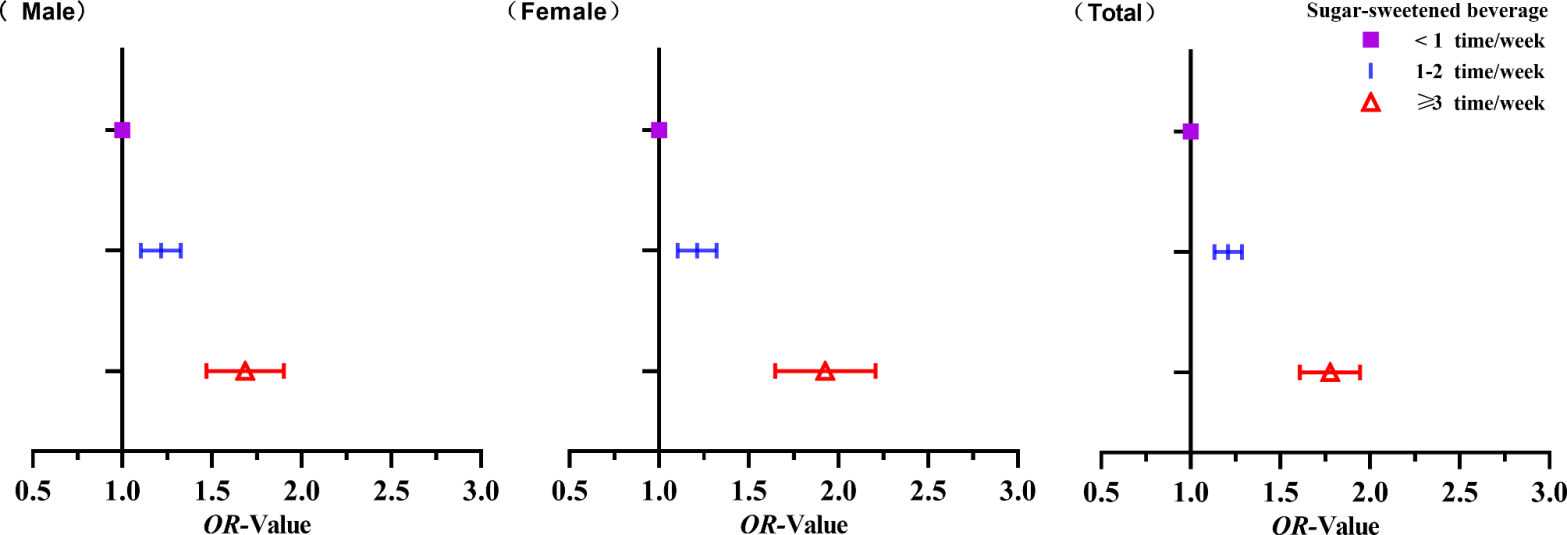
Change in OR values of logistic regression analysis of sugar-sweetened beverage consumption frequency and muscle strength index among Chinese adolescents (Model 2)

Table 3 shows that whether (Yes = 1, No = 0) the muscle strength index was lower was used as the dependent variable. The frequency of sugar-sweetened beverage consumption (< 1 time/week = 1, 1–2 times/week = 2, ≥ 3 times/week = 3) in Chinese adolescents was used as the independent variable. Multiple logistic regression analysis was conducted by adjusting for relevant confounders. Crude models were not adjusted for relevant confounding variables, and only the association between sugar-sweetened beverage consumption frequency and muscle strength was analyzed. Model 1 adjusted for age and parental education. Model 2 further adjusted for school commuting patterns, snack intake, duration of physical activity, and BMI based on Model 1. The analysis showed that, overall, Chinese adolescents who consumed sugary drinks 1–2 times/week (*OR* = 1.207, 95% *CI*:1.132–1.287) and sugary drinks ≥ 3 times/week (*OR* = 1.771, 95% *CI*:1.611–1.947) were positively associated with lower muscle strength compared to those who consumed sugary drinks < 1 time/week (*P* < 0.01). The same trend was found for boys and girls. It is suggested that the higher the consumption of sugar-sweetened beverage among Chinese adolescents, the lower the muscle strength, and the negative correlation exists between the two. The changes of OR values are shown in Fig. 2.

**Table 3:** Logistic regression analysis of sugar-sweetened beverage consumption and muscle strength index among Chinese adolescents (n = 25,893)

Sugar-sweetened beverage	*OR*(95% *CI*)		
	Crude model	Model 1	Model 2
**Male**			
<1 times/week	1.000	1.000	1.000
1–2 times/week	1.201(1.097 ~ 1.314) ^a^	1.195(1.092 ~ 1.308) ^a^	1.211(1.105 ~ 1.328) ^a^
≥ 3 times/week	1.641(1.455 ~ 1.851) ^a^	1.649(1.461 ~ 1.860) ^a^	1.675(1.473 ~ 1.904) ^a^
**Female**			
<1 times/week	1.000	1.000	1.000
1–2 times/week	1.157(1.059 ~ 1.263) ^a^	1.154(1.057 ~ 1.261) ^a^	1.208(1.104 ~ 1.322) ^a^
≥ 3 times/week	1.712(1.493 ~ 1.962) ^a^	1.714(1.495 ~ 1.966) ^a^	1.913(1.654 ~ 2.213) ^a^
**Total**			
<1 times/week	1.000	1.000	1.000
1–2 times/week	1.176(1.105 ~ 1.253) ^a^	1.171(1.100 ~ 1.247) ^a^	1.207(1.132 ~ 1.287) ^a^
≥ 3 times/week	1.657(1.515 ~ 1.813) ^a^	1.662(1.520 ~ 1.818) ^a^	1.771(1.611 ~ 1.947) ^a^

Note: ^a^, *p* <0.01. Crude models were not adjusted for relevant confounding variables, and only the association between sugar-sweetened beverage consumption and muscle strength was analyzed. Model 1 adjusted for age and parental education. Model 2 adjusted for school commuting pattern, snack intake, physical activity duration, and BMI based on model 1

## Discussion

This study found that the proportions of Chinese adolescents who consumed sugar-sweetened beverage ≥ 3 times/week, 1–2 times/week, and < 1 time/week were 12.23%, 52.79%, and 34.98%, respectively. This result indicates that more than 60% of adolescents in China consume sugary beverages more than once a week or more, indicating that adolescents are facing serious sugary beverage control efforts. However, several studies at home and abroad have found that cross-sectional comparisons between the results of different studies are not possible due to differences in the definition of sugary beverages and the frequency division of the survey, and the amount of consumption [33, 34]. However, overall it can be shown that Chinese adolescents sugar-sweetened beverage consumption is seriously overdosed and face a serious situation. A survey on Chinese adolescents shows that Chinese adolescents aged 6–17 consume about 193.8 g of sugar-sweetened beverages per day, which poses a serious threat to physical and mental health [35]. Studies have also confirmed that 17% of the energy of American adolescents comes from added sugar, with nearly 50% coming from sugary drinks, and that nearly 20% of the group consumes 620 kcal of added sugar per day, with half coming from sugary drinks, posing many health risks [36]. A number of studies have confirmed that excessive consumption of sugar-sweetened beverage among adolescents has led to an increase in various diseases, such as increased overweight and obesity, decreased cognitive function, decreased academic performance, increased chronic diseases, increased dental caries and digestive diseases, and decreased physical fitness levels, which pose serious threats to their physical and mental health [37–41].

Several studies have confirmed that adolescent muscle strength is strongly associated with physical and mental health during adolescence and future health in adulthood [42]. Studies have shown that poor muscle strength is also associated with chronic cardiovascular disease, hypertension, diabetes, and spinal curvature, which seriously affects adolescents’ health and academic performance and has a negative impact on them [43–45]. However, there are more factors that influence muscle strength in adolescents. In addition to the level of exercise, there is a strong correlation with eating behavior. Studies have shown that obese individuals have relatively low muscle strength and especially lower levels of explosive power [46]. The present study showed that the proportion of Chinese adolescents with higher frequency of sugar-sweetened beverage consumption with muscle index ≥ P25 was lower, only 11.1%. This result suggests that Chinese adolescents with higher frequency of sugar-sweetened beverage consumption have relatively lower muscle strength.

This study also found that Chinese adolescents with excessive sugar-sweetened beverage consumption had relatively lower levels of muscle strength. In addition, further logistic regression analysis showed that Chinese adolescents who consumed sugar-sweetened beverages 1–2 times/week and ≥ 3 times/week were positively associated with lower muscle strength compared with those who consumed sugar-sweetened beverages < 1 time/week, and this relationship did not change by gender. This suggests that sugar-sweetened beverage consumption is associated with adolescent muscle strength and has a negative impact on it. The reason for this is that excessive sugar-sweetened beverage consumption leads to the development of obesity and weight gain affects lower limb muscle strength-based standing long jump performance. The study confirmed that compared to normal weight adolescents, overweight and obese adolescents have a lower level of physical fitness, one of which is the standing long jump, and therefore need to overcome their greater weight resistance to complete the test, which inevitably has a negative impact on their performance [47]. Studies have also confirmed that sugary beverages, which are readily available to adolescents, are the biggest cause of obesity because excessive sugar-sweetened beverage consumption can lead to disorders of the gastrointestinal tract and metabolic diseases, resulting in excessive caloric intake and thus causing obesity to occur [48]. Excessive sugar-sweetened beverage consumption induces cellular autophagy and telomere shortening, leading to decreased muscle function. Studies have confirmed that excessive consumption of sugar-sweetened beverages leads to an increase in body sugar, which induces cellular autophagy and telomere shortening in humans, accelerating cellular senescence and apoptosis, thereby reducing muscle mass and thus affecting muscle strength and function [49]. A study of adolescents showed that after 30 days of consuming sugary drinks, adolescents gained weight and muscle fat, and insulin and serum glucose levels continued to rise, as did muscle triglyceride and interleukin-6 levels, resulting in a metabolic stress response that led to much higher than normal activation of muscle cell autophagy, causing a decrease in muscle function and strength [50].

However, it is of concern that previous studies on the effects of sugary drinks and muscle strength were higher in boys than in girls, i.e., the risk values were higher in boys than in girls, and this result is not consistent with the present study [50]. The reason may be because, the different studies investigated the consumption of sugary drinks differently, plus the different age and ethnic composition of the groups, which may also be an important reason for the inconsistent results of different studies. For whether there are gender differences in the association between sugary drinks and muscle strength, further investigation and analysis are needed in future studies.

The present study also has some limitations. On the one hand, this study is a cross-sectional study, which can only understand the correlation between sugary drinks and muscle strength, but not the causal relationship between them. On the other hand, this study only investigated the frequency of sugar-sweetened beverage consumption, and the amount and type of sugar-sweetened beverage consumption is not clear, so further investigation and analysis are needed in future studies. However, there are some strengths of this study. First, to the best of our knowledge, this is the first investigation and analysis of the association between sugar-sweetened beverage consumption and muscle strength in Chinese adolescents, which can help in the intervention and improvement of muscle strength in Chinese adolescents. Second, the survey sample involved different regions of China, and the sample size was relatively large and representative.

## Conclusion

Sugar-sweetened beverage consumption was more common among Chinese adolescents, and there was a negative association between sugar-sweetened beverage consumption and muscle strength. In the future, we should strengthen adolescent health education, improve health knowledge related to sugar-sweetened beverages, and reduce sugar-sweetened beverage consumption. In addition, physical exercise should be strengthened to maintain good exercise and dietary behavior habits to promote the continuous improvement of muscle strength and ensure healthy physical and mental development.

## Data Availability

All data generated or analysed during this study are included in supplementary information files.

## Abbreviations

SSB: Sugar-sweetened beverages
SD: Standard Deviation
